# (Di-2-pyridyl­amine)­(methanol)sulfato­copper(II)

**DOI:** 10.1107/S1600536810038675

**Published:** 2010-10-02

**Authors:** Paul DeBurgomaster, Jon Zubieta

**Affiliations:** a Department of Chemistry, Syracuse University, Syracuse, New York 13244, USA

## Abstract

The title complex, [Cu(SO_4_)(C_10_H_9_N_3_)(CH_3_OH)], is a mononuclear species with the Cu^II^ ion in a Jahn–Teller-distorted ‘4 + 1’ square-pyramidal geometry. The basal plane is defined by the pyridyl N-atom donors of the bipyridyl­amine (bpa) ligand and two O-atom donors of the sulfate ligand. The coordination geometry is completed by the axial coordination of a methanol O-atom donor. The axial bond length displays the usual elongation: Cu—O(axial) = 2.168 (2), Cu—O(basal) = 2.016 (2) (average) and Cu—N(basal) = 1.951 (3) Å (average). In the crystal structure, the complex mol­ecules are linked through N—H⋯O and O—H⋯O hydrogen bonds into chains along [100].

## Related literature

For structures of other copper-bis­(2-pyrid­yl)amine complexes, see: Fischer & Bau (1977 ▶); Kavounis *et al.* (1999 ▶); Youngme *et al.* (2005 ▶). For solvatothermal chemistry of compounds containing copper-bis­(2-pyrid­yl)amine subunits, see: DeBurgo­master *et al.* (2010 ▶). For structural chemistry of the related tridentate ligand bis­(2-pyridyl­meth­yl)amine, see: Bartholomä *et al.* (2010*a* ▶, *b* ▶,*c* ▶,*d* ▶,*e* ▶). For copper–pyridyl subunits in the design of organic–inorganic hybrid materials, see: Armatas *et al.* (2005 ▶); Chesnut *et al.* (1999 ▶); Hagrman *et al.* (1999 ▶).
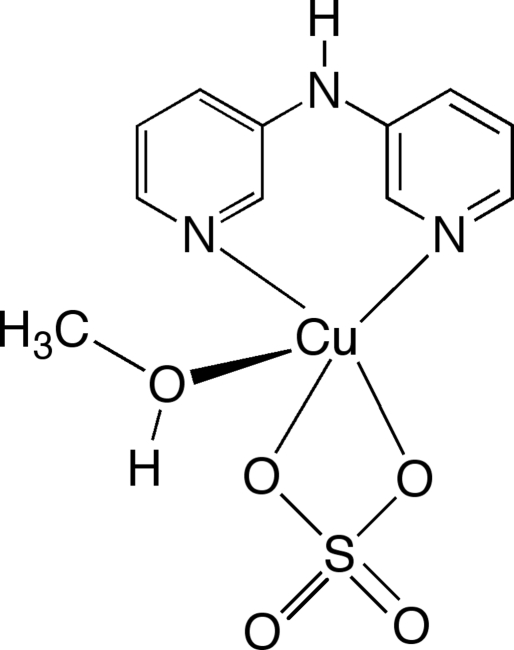


## Experimental

### 

#### Crystal data

[Cu(SO_4_)(C_10_H_9_N_3_)(CH_4_O)]
*M* *_r_* = 362.84
Monoclinic, 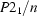

*a* = 7.1403 (10) Å
*b* = 10.7361 (15) Å
*c* = 17.798 (3) Å
β = 92.185 (3)°
*V* = 1363.4 (3) Å^3^
*Z* = 4
Mo *K*α radiation
μ = 1.78 mm^−1^
*T* = 90 K
0.30 × 0.15 × 0.07 mm

#### Data collection

Bruker APEX CCD area-detector diffractometer
Absorption correction: multi-scan (*SADABS*; Bruker, 1998 ▶) *T*_min_ = 0.617, *T*_max_ = 0.886
13215 measured reflections
3308 independent reflections
3119 reflections with *I* > 2σ(*I*)
*R*_int_ = 0.033

#### Refinement

*R*[*F* ^2^ > 2σ(*F* ^2^)] = 0.052
*wR*(*F* ^2^) = 0.109
*S* = 1.26
3308 reflections
192 parameters
H-atom parameters constrained
Δρ_max_ = 0.79 e Å^−3^
Δρ_min_ = −0.75 e Å^−3^

### 

Data collection: *SMART* (Bruker, 1998 ▶); cell refinement: *SAINT* (Bruker, 1998 ▶); data reduction: *SAINT*; program(s) used to solve structure: *SHELXS97* (Sheldrick, 2008 ▶); program(s) used to refine structure: *SHELXL97* (Sheldrick, 2008 ▶); molecular graphics: *CrystalMaker* (Palmer, 2006 ▶); software used to prepare material for publication: *SHELXTL* (Sheldrick, 2008 ▶).

## Supplementary Material

Crystal structure: contains datablocks I, global. DOI: 10.1107/S1600536810038675/hg2716sup1.cif

Structure factors: contains datablocks I. DOI: 10.1107/S1600536810038675/hg2716Isup2.hkl

Additional supplementary materials:  crystallographic information; 3D view; checkCIF report

## Figures and Tables

**Table 1:** Hydrogen-bond geometry (Å, °)

*D*—H⋯*A*	*D*—H	H⋯*A*	*D*⋯*A*	*D*—H⋯*A*
N2—H*N*2⋯O3^i^	0.90	1.97	2.854 (3)	169
O5—H*O*5⋯O3^ii^	0.89	1.82	2.700 (3)	168

Symmetry codes: (i) 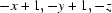; (ii) 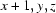.

**Fig. 1.**
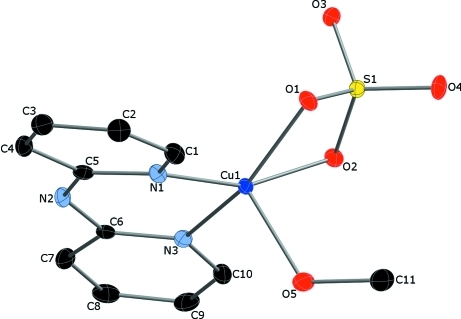
An ORTEP view of the structure of the title complex, showing displacement ellipsoids at the 50% probability level and the atom-labeling scheme. The pyridyl group hydrogen atoms have been omitted for clarity.

**Fig. 2.**
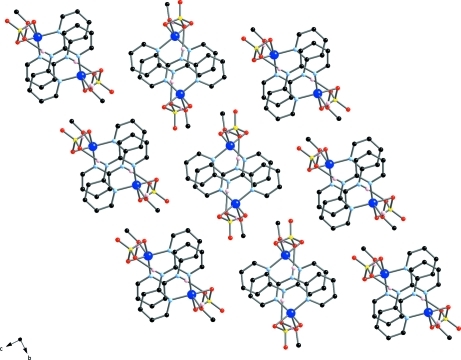
A view of the packing in the bc plane.

**Fig. 3.**
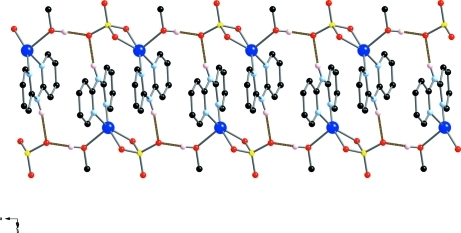
A view of the hydrogen-bonded double chains running parallel to [100]. The hydrogen bonds are shown as red and green multiband cylinders.

## supplementary crystallographic information

### Comment

In the course of our investigations of the design of materials constructed from metal oxide nodes linked through or decorated with copper-pyridyl subunits (Armatas *et al.* (2005); Chesnut *et al.* (1999); Hagrman *et al.* (1999)), we prepared and investigated a series of dipodal ligands with bis(2-pyridylmethyl)amine termini (Bartholomä *et al.* (2010a,b,c,d,e). Since this ligand acts as a tridentate donor, the structural consequences of introducing an analogous bidentate ligand, such as bis(2-pyridyl)amine were of interest in expanding the structural data base. Representative examples of copper-bis(2-pyridyl)amine complexes have been reported (Fischer & Bau (1977); Kavounis *et al.* (1999); Youngme *et al.* (2005)), as well as {Cu(bpa)}^2+^ subunits in metal oxide complexes (DeBurgomaster *et al.* (2010)). As shown in Fig. 1, the mononuclear complex exhibits copper(II) sites in a distorted '4 + 1' square pyramidal geometry. The basal plane is defined by the pyridyl nitrogen donors of the bis(2-pyridyl)amine ligand and two sulfato oxygen donors, while the apical position is occupied by the oxygen donor of the methanol ligand. The bond lengths demonstrate the lengthening of the axial Cu—O bond with respect to the bonds in the basal plane: Cu—N1, 1.946 (3) Å; Cu—N2, 1.955 (3) Å; Cu—O1, 2.004 (2) Å; Cu—O2, 2.027 (2) Å; Cu—O5, 2.168 (2) Å. The structure is stabilized by intermolecular hydrogen-bonding between the amine N—H group and a pendant sulfate oxygen and between the methanol O—H group and the pendant sulfate oxygen (Fig. 2). This results in a one-dimensional hydrogen-bonded double chain parallel to the [100] direction (Fig. 3).

### Experimental

Synthesis of [Cu(SO_4_)(C_10_H_9_N_3_)(CH_3_OH)]. A solution of Cu(SO_4_).5H2O (0.250 g, 1.0 mmol) and bis(2-pyridyl)amine (0.171 g, 1.0 mmol) in 10 ml of methanol was heated to 75° C for 48 h (initial and final pH, 4.0). Blue crystals of the product were isolated in 25% yield. Anal. Calcd. for C_11_H_13_CuN_3_O_5_S: C, 36.4; H, 3.58; N, 11.6. Found: C, 36.2; H, 3.69; N, 11.5.

### Refinement

Pyridyl hydrogen atoms were discernable in the difference Fourier map. The hydrogen atoms were placed in calculated positions with C—H = 0.95 Å and included in the riding model approximation with *U*_iso_(H) = 1.2*U*_eq_(C). The amine hydrogen atom and the hydrogen associated with the oxygen of the methanol molecule were also found on the difference Fourier map. These were included in the coordinate riding approximation with *U*_iso_(H) free to vary.

### Crystal data

**Table:**

[Cu(SO_4_)(C_10_H_9_N_3_)(CH_4_O)]	*F*(000) = 740
*M**_r_* = 362.84	*D*_x_ = 1.768 Mg m^−^^3^*D*_m_ = 1.75 (2) Mg m^−^^3^*D*_m_ measured by flotation
Monoclinic, *P*2_1_/*n*	Mo *K*α radiation, λ = 0.71073 Å
Hall symbol: -P 2yn	Cell parameters from 4522 reflections
*a* = 7.1403 (10) Å	θ = 3.0–28.3°
*b* = 10.7361 (15) Å	µ = 1.78 mm^−^^1^
*c* = 17.798 (3) Å	*T* = 90 K
β = 92.185 (3)°	Plate, green
*V* = 1363.4 (3) Å^3^	0.30 × 0.15 × 0.07 mm
*Z* = 4	

### Data collection

**Table:**

Bruker APEX CCD area-detector diffractometer	3308 independent reflections
Radiation source: fine-focus sealed tube	3119 reflections with *I* > 2σ(*I*)
graphite	*R*_int_ = 0.033
Detector resolution: 512 pixels mm^-1^	θ_max_ = 28.1°, θ_min_ = 3.0°
φ and ω scans	*h* = −9→9
Absorption correction: multi-scan (*SADABS*; Bruker, 1998)	*k* = −14→13
*T*_min_ = 0.617, *T*_max_ = 0.886	*l* = −23→23
13215 measured reflections	

### Refinement

**Table:**

Refinement on *F*^2^	Primary atom site location: structure-invariant direct methods
Least-squares matrix: full	Secondary atom site location: difference Fourier map
*R*[*F*^2^ > 2σ(*F*^2^)] = 0.052	Hydrogen site location: inferred from neighbouring sites
*wR*(*F*^2^) = 0.109	H-atom parameters constrained
*S* = 1.26	*w* = 1/[σ^2^(*F*_o_^2^) + (0.0374*P*)^2^ + 3.5736*P*] where *P* = (*F*_o_^2^ + 2*F*_c_^2^)/3
3308 reflections	(Δ/σ)_max_ < 0.001
192 parameters	Δρ_max_ = 0.79 e Å^−^^3^
0 restraints	Δρ_min_ = −0.75 e Å^−^^3^

### Special details

**Table:**

Geometry. All e.s.d.'s (except the e.s.d. in the dihedral angle between two l.s. planes) are estimated using the full covariance matrix. The cell e.s.d.'s are taken into account individually in the estimation of e.s.d.'s in distances, angles and torsion angles; correlations between e.s.d.'s in cell parameters are only used when they are defined by crystal symmetry. An approximate (isotropic) treatment of cell e.s.d.'s is used for estimating e.s.d.'s involving l.s. planes.
Refinement. Refinement of *F*^2^ against ALL reflections. The weighted *R*-factor *wR* and goodness of fit *S* are based on *F*^2^, conventional *R*-factors *R* are based on *F*, with *F* set to zero for negative *F*^2^. The threshold expression of *F*^2^ > σ(*F*^2^) is used only for calculating *R*-factors(gt) *etc*. and is not relevant to the choice of reflections for refinement. *R*-factors based on *F*^2^ are statistically about twice as large as those based on *F*, and *R*- factors based on ALL data will be even larger.

### Fractional atomic coordinates and isotropic or equivalent isotropic displacement parameters (Å^2^)

**Table:**

	*x*	*y*	*z*	*U*_iso_*/*U*_eq_	
Cu1	0.64877 (5)	0.27906 (3)	0.05295 (2)	0.00870 (12)	
S1	0.37436 (10)	0.16887 (7)	0.11547 (4)	0.00900 (16)	
O1	0.4394 (3)	0.1566 (2)	0.03641 (12)	0.0116 (4)	
O2	0.5310 (3)	0.2441 (2)	0.15261 (13)	0.0118 (5)	
O3	0.2005 (3)	0.2444 (2)	0.11458 (13)	0.0131 (5)	
O4	0.3483 (3)	0.0501 (2)	0.15113 (13)	0.0138 (5)	
O5	0.8729 (3)	0.1433 (2)	0.06432 (14)	0.0174 (5)	
HO5	0.9887	0.1690	0.0770	0.029 (12)*	
N1	0.6684 (4)	0.3176 (2)	−0.05332 (15)	0.0100 (5)	
N2	0.7276 (4)	0.5328 (2)	−0.03312 (15)	0.0111 (5)	
HN2	0.7638	0.5982	−0.0606	0.017 (10)*	
N3	0.7710 (4)	0.4339 (2)	0.08540 (15)	0.0105 (5)	
C1	0.6369 (4)	0.2227 (3)	−0.10237 (18)	0.0134 (6)	
H1	0.6295	0.1404	−0.0832	0.016*	
C2	0.6153 (5)	0.2405 (3)	−0.17841 (19)	0.0144 (6)	
H2	0.5978	0.1717	−0.2115	0.017*	
C3	0.6197 (5)	0.3619 (3)	−0.20600 (19)	0.0152 (7)	
H3	0.5984	0.3774	−0.2582	0.018*	
C4	0.6550 (4)	0.4588 (3)	−0.15736 (19)	0.0129 (6)	
H4	0.6601	0.5419	−0.1755	0.015*	
C5	0.6835 (4)	0.4337 (3)	−0.08069 (18)	0.0103 (6)	
C6	0.7789 (4)	0.5367 (3)	0.04263 (18)	0.0106 (6)	
C7	0.8364 (5)	0.6517 (3)	0.07262 (19)	0.0147 (6)	
H7	0.8393	0.7236	0.0415	0.018*	
C8	0.8885 (5)	0.6594 (3)	0.1477 (2)	0.0169 (7)	
H8	0.9272	0.7367	0.1691	0.020*	
C9	0.8838 (5)	0.5519 (3)	0.19230 (19)	0.0166 (7)	
H9	0.9213	0.5546	0.2441	0.020*	
C10	0.8242 (4)	0.4435 (3)	0.15956 (18)	0.0138 (6)	
H10	0.8194	0.3709	0.1899	0.017*	
C11	0.8502 (5)	0.0179 (3)	0.0901 (2)	0.0208 (8)	
H11A	0.9712	−0.0252	0.0903	0.031*	
H11B	0.7603	−0.0258	0.0565	0.031*	
H11C	0.8036	0.0189	0.1412	0.031*	

### Atomic displacement parameters (Å^2^)

**Table:**

	*U*^11^	*U*^22^	*U*^33^	*U*^12^	*U*^13^	*U*^23^
Cu1	0.00907 (19)	0.00779 (19)	0.00915 (19)	−0.00177 (14)	−0.00090 (13)	0.00045 (14)
S1	0.0095 (3)	0.0083 (3)	0.0091 (3)	−0.0007 (3)	−0.0005 (3)	0.0008 (3)
O1	0.0126 (11)	0.0117 (11)	0.0104 (10)	−0.0052 (8)	−0.0002 (8)	−0.0010 (9)
O2	0.0124 (11)	0.0116 (11)	0.0114 (10)	−0.0031 (8)	−0.0018 (8)	−0.0003 (8)
O3	0.0119 (11)	0.0087 (10)	0.0187 (12)	0.0012 (8)	0.0000 (9)	0.0031 (9)
O4	0.0200 (12)	0.0067 (10)	0.0145 (11)	−0.0010 (9)	−0.0005 (9)	0.0022 (9)
O5	0.0122 (11)	0.0119 (11)	0.0277 (13)	−0.0005 (9)	−0.0028 (10)	0.0031 (10)
N1	0.0094 (12)	0.0096 (12)	0.0112 (12)	−0.0001 (10)	0.0022 (9)	−0.0007 (10)
N2	0.0131 (13)	0.0064 (12)	0.0138 (13)	−0.0013 (10)	−0.0010 (10)	0.0018 (10)
N3	0.0093 (12)	0.0098 (12)	0.0122 (13)	−0.0012 (10)	−0.0015 (10)	0.0004 (10)
C1	0.0146 (15)	0.0093 (14)	0.0163 (16)	−0.0012 (12)	0.0010 (12)	−0.0021 (12)
C2	0.0167 (16)	0.0125 (15)	0.0139 (15)	−0.0004 (12)	0.0008 (12)	−0.0046 (12)
C3	0.0142 (15)	0.0210 (17)	0.0107 (15)	0.0017 (13)	0.0026 (12)	0.0012 (13)
C4	0.0139 (15)	0.0089 (14)	0.0160 (16)	0.0001 (12)	0.0020 (12)	0.0019 (12)
C5	0.0067 (13)	0.0112 (14)	0.0132 (15)	0.0016 (11)	0.0013 (11)	−0.0023 (12)
C6	0.0063 (13)	0.0124 (15)	0.0132 (15)	0.0002 (11)	0.0004 (11)	0.0004 (12)
C7	0.0153 (16)	0.0093 (15)	0.0191 (16)	0.0007 (12)	−0.0033 (12)	0.0016 (13)
C8	0.0139 (16)	0.0155 (16)	0.0211 (17)	−0.0030 (13)	−0.0031 (13)	−0.0071 (14)
C9	0.0130 (15)	0.0227 (18)	0.0137 (16)	−0.0006 (13)	−0.0044 (12)	−0.0014 (13)
C10	0.0125 (15)	0.0166 (16)	0.0119 (15)	−0.0030 (12)	−0.0022 (12)	0.0028 (12)
C11	0.0171 (17)	0.0091 (16)	0.036 (2)	0.0005 (13)	−0.0048 (15)	0.0021 (14)

### Geometric parameters (Å, °)

**Table:**

Cu1—N1	1.946 (3)	C1—H1	0.9500
Cu1—N3	1.955 (3)	C2—C3	1.394 (5)
Cu1—O1	2.004 (2)	C2—H2	0.9500
Cu1—O2	2.027 (2)	C3—C4	1.370 (5)
Cu1—O5	2.168 (2)	C3—H3	0.9500
S1—O4	1.439 (2)	C4—C5	1.398 (5)
S1—O3	1.482 (2)	C4—H4	0.9500
S1—O1	1.504 (2)	C6—C7	1.401 (4)
S1—O2	1.511 (2)	C7—C8	1.375 (5)
O5—C11	1.434 (4)	C7—H7	0.9500
O5—HO5	0.8923	C8—C9	1.402 (5)
N1—C5	1.343 (4)	C8—H8	0.9500
N1—C1	1.355 (4)	C9—C10	1.363 (5)
N2—C6	1.384 (4)	C9—H9	0.9500
N2—C5	1.389 (4)	C10—H10	0.9500
N2—HN2	0.8988	C11—H11A	0.9800
N3—C6	1.343 (4)	C11—H11B	0.9800
N3—C10	1.363 (4)	C11—H11C	0.9800
C1—C2	1.369 (5)		
			
N1—Cu1—N3	93.33 (11)	C1—C2—C3	118.4 (3)
N1—Cu1—O1	94.47 (10)	C1—C2—H2	120.8
N3—Cu1—O1	157.45 (10)	C3—C2—H2	120.8
N1—Cu1—O2	159.62 (10)	C4—C3—C2	119.6 (3)
N3—Cu1—O2	95.43 (10)	C4—C3—H3	120.2
O1—Cu1—O2	71.02 (9)	C2—C3—H3	120.2
N1—Cu1—O5	98.83 (10)	C3—C4—C5	119.1 (3)
N3—Cu1—O5	102.96 (10)	C3—C4—H4	120.4
O1—Cu1—O5	96.70 (9)	C5—C4—H4	120.4
O2—Cu1—O5	97.08 (9)	N1—C5—N2	120.6 (3)
O4—S1—O3	111.51 (14)	N1—C5—C4	121.5 (3)
O4—S1—O1	112.62 (14)	N2—C5—C4	117.9 (3)
O3—S1—O1	109.03 (13)	N3—C6—N2	120.8 (3)
O4—S1—O2	112.73 (14)	N3—C6—C7	121.8 (3)
O3—S1—O2	108.55 (13)	N2—C6—C7	117.4 (3)
O1—S1—O2	101.91 (13)	C8—C7—C6	119.2 (3)
S1—O1—Cu1	93.46 (11)	C8—C7—H7	120.4
S1—O2—Cu1	92.35 (11)	C6—C7—H7	120.4
C11—O5—Cu1	124.7 (2)	C7—C8—C9	119.3 (3)
C11—O5—HO5	108.9	C7—C8—H8	120.3
Cu1—O5—HO5	119.3	C9—C8—H8	120.3
C5—N1—C1	118.5 (3)	C10—C9—C8	118.3 (3)
C5—N1—Cu1	124.0 (2)	C10—C9—H9	120.8
C1—N1—Cu1	116.7 (2)	C8—C9—H9	120.8
C6—N2—C5	131.5 (3)	C9—C10—N3	123.3 (3)
C6—N2—HN2	115.8	C9—C10—H10	118.3
C5—N2—HN2	109.2	N3—C10—H10	118.3
C6—N3—C10	118.1 (3)	O5—C11—H11A	109.5
C6—N3—Cu1	124.0 (2)	O5—C11—H11B	109.5
C10—N3—Cu1	117.0 (2)	H11A—C11—H11B	109.5
N1—C1—C2	122.8 (3)	O5—C11—H11C	109.5
N1—C1—H1	118.6	H11A—C11—H11C	109.5
C2—C1—H1	118.6	H11B—C11—H11C	109.5
			
O4—S1—O1—Cu1	−130.74 (12)	N1—Cu1—N3—C10	−168.3 (2)
O3—S1—O1—Cu1	104.93 (12)	O1—Cu1—N3—C10	81.6 (4)
O2—S1—O1—Cu1	−9.70 (13)	O2—Cu1—N3—C10	30.1 (2)
N1—Cu1—O1—S1	−157.87 (12)	O5—Cu1—N3—C10	−68.4 (2)
N3—Cu1—O1—S1	−48.0 (3)	C5—N1—C1—C2	−1.6 (5)
O2—Cu1—O1—S1	7.46 (10)	Cu1—N1—C1—C2	168.9 (3)
O5—Cu1—O1—S1	102.68 (12)	N1—C1—C2—C3	−2.2 (5)
O4—S1—O2—Cu1	130.54 (12)	C1—C2—C3—C4	3.4 (5)
O3—S1—O2—Cu1	−105.41 (12)	C2—C3—C4—C5	−0.8 (5)
O1—S1—O2—Cu1	9.58 (13)	C1—N1—C5—N2	−175.8 (3)
N1—Cu1—O2—S1	39.0 (3)	Cu1—N1—C5—N2	14.4 (4)
N3—Cu1—O2—S1	154.08 (12)	C1—N1—C5—C4	4.3 (4)
O1—Cu1—O2—S1	−7.42 (10)	Cu1—N1—C5—C4	−165.4 (2)
O5—Cu1—O2—S1	−102.10 (11)	C6—N2—C5—N1	6.5 (5)
N1—Cu1—O5—C11	−122.5 (3)	C6—N2—C5—C4	−173.6 (3)
N3—Cu1—O5—C11	141.9 (3)	C3—C4—C5—N1	−3.1 (5)
O1—Cu1—O5—C11	−26.9 (3)	C3—C4—C5—N2	177.0 (3)
O2—Cu1—O5—C11	44.7 (3)	C10—N3—C6—N2	179.7 (3)
N3—Cu1—N1—C5	−24.1 (3)	Cu1—N3—C6—N2	−11.3 (4)
O1—Cu1—N1—C5	134.7 (3)	C10—N3—C6—C7	−1.1 (4)
O2—Cu1—N1—C5	91.3 (4)	Cu1—N3—C6—C7	167.9 (2)
O5—Cu1—N1—C5	−127.8 (2)	C5—N2—C6—N3	−8.2 (5)
N3—Cu1—N1—C1	166.0 (2)	C5—N2—C6—C7	172.6 (3)
O1—Cu1—N1—C1	−35.2 (2)	N3—C6—C7—C8	0.8 (5)
O2—Cu1—N1—C1	−78.6 (4)	N2—C6—C7—C8	−180.0 (3)
O5—Cu1—N1—C1	62.3 (2)	C6—C7—C8—C9	0.4 (5)
N1—Cu1—N3—C6	22.6 (3)	C7—C8—C9—C10	−1.2 (5)
O1—Cu1—N3—C6	−87.5 (4)	C8—C9—C10—N3	0.9 (5)
O2—Cu1—N3—C6	−139.0 (3)	C6—N3—C10—C9	0.3 (5)
O5—Cu1—N3—C6	122.4 (2)	Cu1—N3—C10—C9	−169.5 (3)

### Hydrogen-bond geometry (Å, °)

**Table:**

*D*—H···*A*	*D*—H	H···*A*	*D*···*A*	*D*—H···*A*
N2—HN2···O3^i^	0.90	1.97	2.854 (3)	169
O5—HO5···O3^ii^	0.89	1.82	2.700 (3)	168

Symmetry codes: (i) −*x*+1, −*y*+1, −*z*; (ii) *x*+1, *y*, *z*.
